# Imaging changes after breast reconstruction with fat grafting - Retrospective study of 90 breast cancer

**DOI:** 10.12669/pjms.321.9460

**Published:** 2016

**Authors:** Lubna Noor, Helen Rosemarry Reeves, Dileep Kumar, Ous Alozairi, Pudhupalayam Bhaskar

**Affiliations:** 1 Lubna Noor, FRCS, FCPS, EBSQ (Breast Surgery), Specialty Doctor (Breast Surgery), University Hospital of North Tees, Hardwick, Stockton on Tees, TS198PE, UK; 2 Helen Rosemarry Reeves, MSc Diagnostic Imaging, BSc Diagnostic Radiography, Consultant Practitioner, Breast Unit, University Hospital of North Tees, Hardwick, Stockton on Tees, TS198PE, UK; 3 Dileep Kumar, SHO Surgery, University Hospital of North Tees, Hardwick, Stockton on Tees, TS198PE, UK; 4 Ous Alozairi, FRCS, MBCHB, MSc, Consultant Breast and General Surgeon, University Hospital of North Tees, Hardwick, Stockton on Tees, TS198PE, UK; 5 Pudhupalayam Bhaskar, FRCS, MD, Consultant Oncoplastic Breast and General Surgeon, University Hospital of North Tees, Hardwick, Stockton on Tees, TS198PE, UK

**Keywords:** Breast cancer, Breast imaging, Breast reconstruction, Fat grafting. Mammography, FG: Fat grafting, PMR: Post Mastectomy Reconstruction, WLE: Wide Local Excision, BI-RADS: Breast Imaging Reporting and Data system, M: Mammograms, CC: Craniocaudal, MLO: Mediolateral Oblique, MRI: Magnetic Resonance Imaging, FN: Fat Necrosis

## Abstract

**Objective:**

To evaluate the breast imaging changes after fat grafting and its impact on cancer follow up.

**Methods:**

This is a retrospective observational study conducted on patients who underwent fat grafting for breast reconstruction. We reviewed mammographic and ultrasound images of patients. Fisher’s exact test was used to analyze results. The level of significance was set at P < 0.05.

**Results:**

A total of ninety patients with breast cancer had fat grafting. Fifty eight patients for defects following post mastectomy reconstruction and 32 for wide local excision defects. The mean follow up was 37.4 months. Benign lumps were identified in 23/90 cases (25 percent). Mammograms were reported as BI-RADS I in 21/32 cases (72 percent) and BI-RADS II in 8/32 cases (28 percent). BI-RADs III score was reported in two patients on further follow up imaging, both were re-classified as BI-RADS II after biopsy. A total of eight patients (8.9 percent) required biopsy. No local recurrences or new cancers were observed in any patients.

**Conclusion:**

Our study suggests radiological changes after fat grafting are almost always benign with no adverse outcome on cancer follow up.

## INTRODUCTION

Advances in reconstructive breast surgery has improved outcome in breast reconstructions as well as raised the patient’s expectations in restoring breast symmetry. Fat grafting (FG) is a useful adjunct in breast reconstruction to correct defects after wide local excision (WLE) and post mastectomy reconstructions (PMR) patients.

Although fat grafting is a fairly simple procedure but there are few concerns. Firstly grafted fat can cause changes in breast tissue with difficulty in interpretation of imaging and interference with cancer diagnosis.^1^-^3^ Other concern is that local growth factors and adipose derived regenerative cells (ADRC’s) in grafted fat can induce new cancer growth or predispose to a higher risk of cancer recurrence.^4^-^6^

The purpose of this study was to evaluate imaging changes following fat grafting for breast reconstruction its impact on cancer follow-up.

## METHODS

We retrospectively reviewed images and case notes of all patients who underwent fat grafting (FG) for breast reconstruction from September 2008 to June 2015. We divided patients in two groups Group-I had FG for WLE defects and Group-II for PMR defects.

A total of 110 FG procedures were performed in 100 patients during this period including 10 with bilateral procedures. We included 90 patients with fat grafting for secondary breast reconstruction following cancer surgery. Group I included 58 patients (64 percent) with FG for post mastectomy reconstruction defects, TRAM FLAP in 14, LD flap in 15 and implant based reconstruction in 29 patients. Group-II included 32 patients (36 percent) with wide local excision defects correction. All these patients had fat grafting after a minimum of 12 months following adjuvant radiotherapy of cancer treatment (range13 to 29 months). Twenty patients with benign indications for fat grafting were excluded.

In patients with history of WLE, mammography before the FG procedure was noted as M0 and post procedure annual mammography as M1 – M5. For study purposes a consultant practitioner reviewed conventional and digital mammograms with craniocaudal (CC) and mediolateral oblique (MLO) projections and ultrasound images of patients.

The Breast Imaging Reporting and Data System (BI-RADS) was used to record the findings. We used Excel 2010 statistical package. Fisher’s exact test to compare outcome in two groups. The level of significance was set at P< 0.05.

## RESULTS

A total of 90 FG procedures were performed for secondary breast reconstruction following cancer surgery during this period.

**Fig.1:**
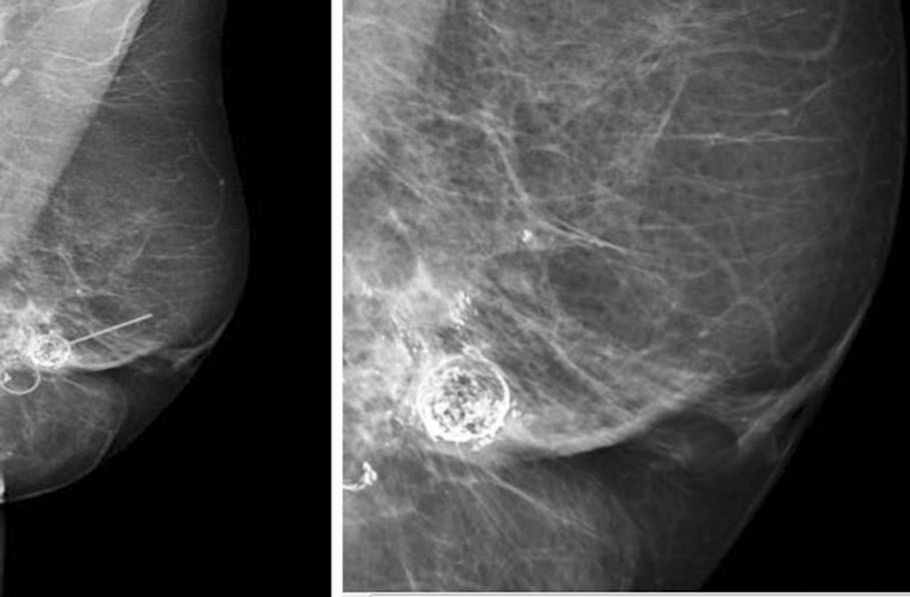
A, Left MLO and magnified views. Mixed fibroglandular parenchyma, scattered calcifications (arrows and circle) with a wide variety of appearances can be seen in the left breast. US scan showed cystic lesions with heterogenous appearance typical of fat necrosis reported as M2.

**Fig.2:**
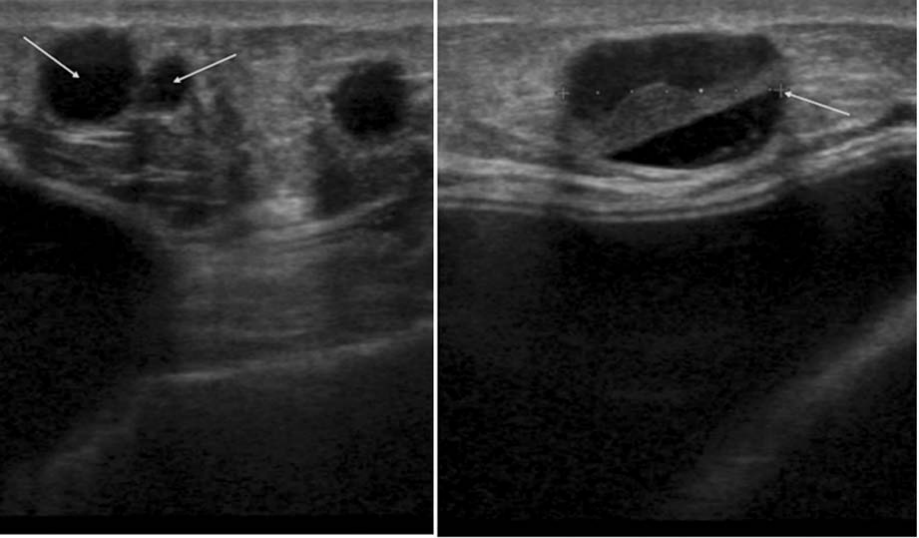
Post mastectomy LD and implant based reconstruction of right breast, had fat grafting (270 ml) in 2009. Clinical manifestation of multiple palpable nodules in second postoperative year. Ultrasound right reconstructed breast showed multiple well defined lesions in the subcutaneous fat consistent with fat necrosis and oil cysts, arrows shows cysts of varied sizes with largest 15 mm reported as U2.

**Fig.3:**
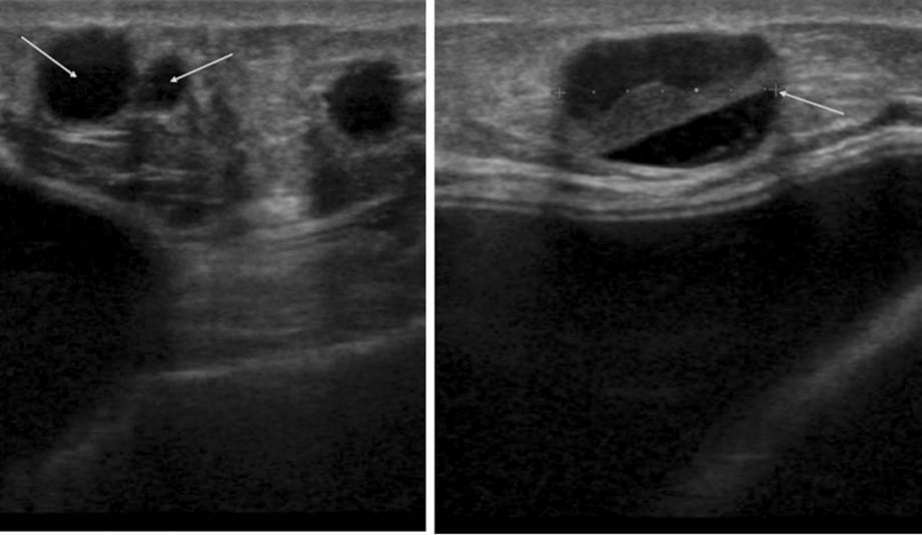
Clinically palpable lump above WLE scar 4 years after grafting (140 ml) in 2008. Mammograms reported as R2. Ultrasound scan showed 14 mm mixed echogenicity lesion reported as likely to be fat necrosis/oil cyst U2, but in view of the solid component ultrasound guided core biopsy performed which confirmed fat necrosis.

**Fig.4:**
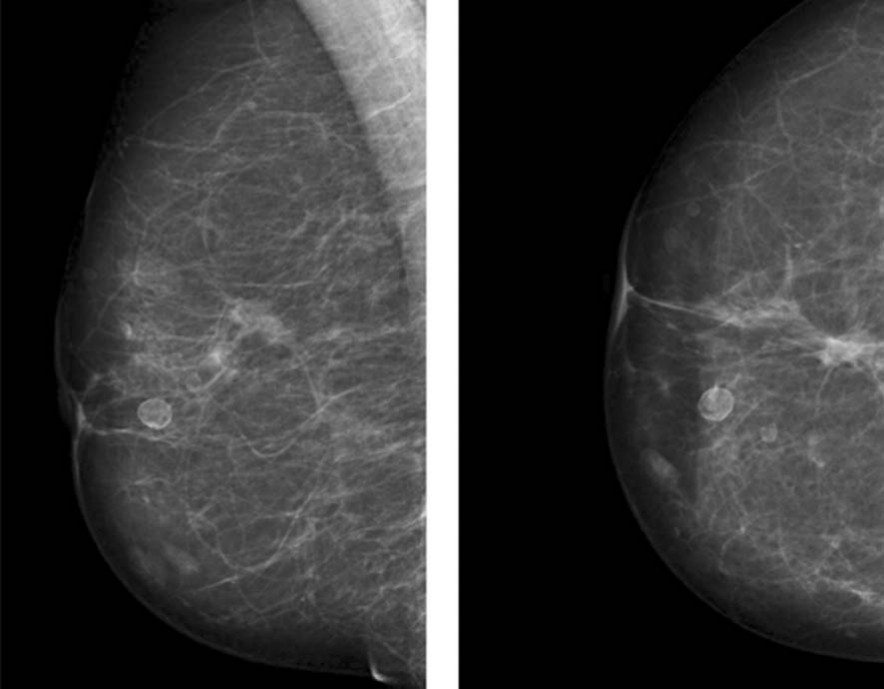
Right MLO & CC views, 3 years after fat grafting (150 ml) in 2009 showing well defined lucent masses surrounded by smooth rims in central and inner breast considered pathognomonic of fat necrosis reported as M2.

All were female and patient ages ranged from 38 to 72 years with median of 53 years. The average volume of fat injected was 154 ml (range 80 ml – 420 ml). The mean follow up following fat grafting was 37.4 months (range 4 to 80 months). Out of 90 FG procedures, clinical lump was reported in 23 (25 percent) patients. These patients underwent further investigations with mammography, ultrasound or biopsy which confirmed benign changes in all symptomatic cases [Table-I]. The predominant imaging changes were fat necrosis and oil cysts [Fig. 1-4]. In more than 80 percent cases, size of fat necrosis was less than 15 mm in size. All were managed conservatively except the one patient. The surgical excision of lump was performed in one case due to patient preference and size of the lump which measured 38 mm.

**Table-I:**
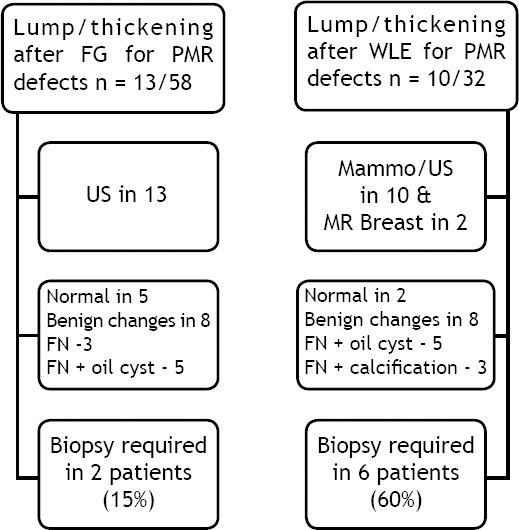
Evaluation of breast cancer patients presented with lumps following fat grafting n=23/90.

We further analyze our data to evaluate clinical lumps and imaging changes after fat grafting for WLE defects versus PMR defects. We observed differences in investigations performed for clinical lumps in two groups. Patients in Group-I required more investigations including MRI of breast and biopsies than those in Group-II. Although no statistical significance reached p-value 0.45 [Table-I].

Image format Table-I: Evaluation of breast cancer patients presented with lumps following fat grafting n=23/90.

**Fig.5:**
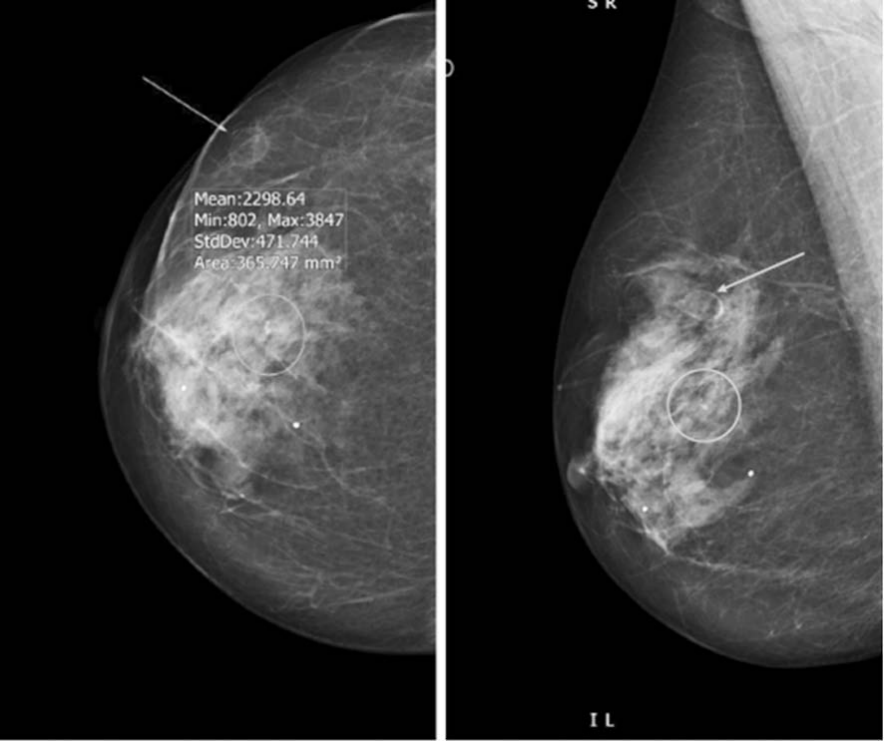
Right MLO and CC projections, 4 years following fat grafting (140 ml). New 5 mm indeterminate calcification (circle) and 9 mm well defined solid/cystic mass (arrow) on n US, BIRADS III. Biopsy of the area confirmed benign calcifications and fat necrosis.

**Fig.6:**
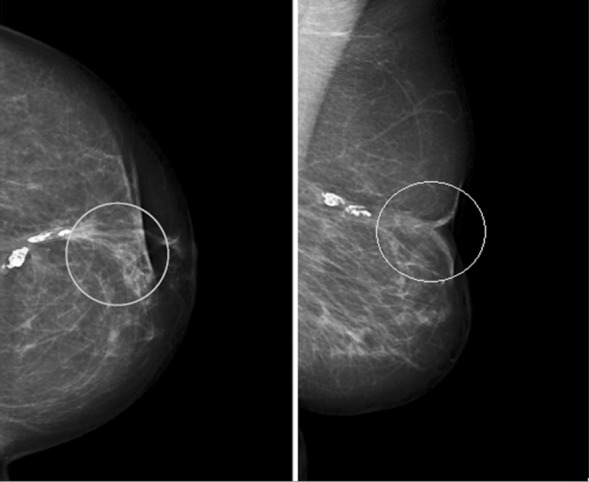
Left CC & MLO projections 2 years following fat grafting (150 ml) in 2011 for left breast WLE defect. Mixed fibro glandular parenchyma, coarse calcification consistent with fat necrosis and an area of suspicious granular calcification (circle) reported as BIRADS III. Stereotactic biopsy confirmed benign calcifications and fat necrosis.

The 58 patients with post mastectomy reconstruction did not have any regular imaging. In 32 eligible patients for bilateral mammography, 29 patients had mammography after the procedure. The average time elapsed between surgery and the first postoperative mammograms was 11 months(6 to 15 months). First post fat grafting mammograms (M-1) were reported as BI-RADS I in 21 cases (72 percent) and BI-RADS II in eight cases (28 percent) [Table-II]. The mammographic findings included oil cysts, micro calcifications, coarse calcifications, focal masses and areas of increased opacity. The predominant finding was fat necrosis with wide variety of benign appearance on imaging. No higher BI-RAD scores were reported. Further follow up imaging reported BI-RADS III score in two cases, at two years and four years after fat grafting [Fig. 5 and 6]. These patients underwent further biopsy confirmation and reclassified as BI-RADS II.

**Table-II:** Post fat grafting yearly mammographic findings in cancer patients.

	BIRADS I	BIRADS II	BIRADS III	BIRADS IV/V
M-0 (n=32)	27	5	0	0
M-1(n=29)	21	8	0	0
M-2 (n=23)	14	8	1	0
M-3(n=13)	10	2	1	0
M-4 (n=11)	9	2	0	0
M-5 (n=7)	6	1	0	0

A total of 8 patients (8.9 percent) needed biopsy for the evaluation of clinical lumps or imaging changes after fat grafting procedure. US guided biopsy was performed in 5, stereotactic biopsy in 2 and open surgical biopsy in 1 patient. The biopsy confirmed benign changes in all of these patients. Almost all, 7 out of 8 biopsies were performed in first few years of study. In the last four years, only one patient required biopsy. No local recurrences or new cancers were reported in any patient during the study period.

## DISCUSSION

Fat grafting, like any other surgical intervention, could lead to alterations in breast tissue and changes in imaging. A wide spectrum of mammographic changes following fat grafting has been reported in the literature, which ranges from benign looking lipid cysts to findings suspicious for malignancy such as clustered micro-calcifications, speculated areas of increased opacity and focal masses.^7^-^9^

A knowledge of the mammographic and ultrasound changes after fat grafting and evolution patterns of fat necrosis are helpful in differentiating benign changes from those associated with breast cancer.

Published literature has reported no increase in the risk of imaging abnormalities or interference in cancer detection following fat grafting to the breast.^10^,^11^ Rubin et al compared mammographic changes after fat transfer with reduction mammoplasty and reported no significant difference in oil cysts or micro-calcifications with fewer biopsies after fat grafting to the breast than after reduction mammoplasty.^12^ In our study, 28 percent benign imaging changes after FG supports reported literature.

The risk of local recurrence is reported to be similar in breast cancer patients with or without FG.^13^ The largest series^10^ of 880 fat grafting procedures to the breast over 10 years showed that procedure is safe and effective with no increased rate of recurrence or new cancer development. Recently published studies and meta-analysis have also established the oncological safety of fat grafting in breast cancer patients.^14^,^15^ Our findings also support the safety of fat grafting in breast cancer patients, with no increased risk of cancer occurrence or local recurrence.

We observed a decreasing trend in the number of biopsies required with the need of only one biopsy in the last three years. This could be due to increase in experience of our radiologists and familiarity to recognize these benign imaging changes following FG.

The role of contrast-enhanced spectral mammography (CESM) with extremely high negative predictive value for breast cancer has been reported in the literature.^16^,^17^ Though we do not have facilities to perform contrast enhanced mammography at our institute but in our opinion one should consider this in the future, especially in difficult cases with micro calcification to reduce the need for tissue diagnosis.

Although in theory changes in breast tissue and imaging after FG can cause difficulty in cancer follow up. But in our experience these changes are almost always benign and could easily be differentiated from those associated with cancer recurrence. We understand the limitation of our study considering retrospective nature and small number of patients and we suggest our results should be confirmed in larger series to ensure reproducibility and improve patient safety.

## CONCLUSION

Our study suggests radiographic changes following fat grafting are almost always benign. Follow-up of breast reconstructed with fat grafting is not difficult. No local recurrences or new cancers were observed in our patients following fat grafting.
